# Pseudoarthrosis of the Distal Humerus in Pediatric Osteogenesis Imperfecta Patients: A Case Series and Literature Review

**DOI:** 10.7759/cureus.58991

**Published:** 2024-04-25

**Authors:** Boaz Laor, Sofia Addab, Chantal Janelle, Reggie C Hamdy

**Affiliations:** 1 Orthopedic Surgery, Shriners Hospitals for Children, Montreal, CAN

**Keywords:** non-union, pediatric orthopedics, functional outcome, distal humerus, osteogenesis imperfecta

## Abstract

Osteogenesis imperfecta (OI) is a rare skeletal disorder that increases a patient’s susceptibility to bone fracture. One complication commonly associated with fractures in this population is the occurrence of non-union leading to pseudoarthrosis. In this case series, three cases of non-union of the distal humerus leading to pseudoarthrosis in the pediatric OI population are presented. One case presents a successful attempt at treatment, one case presents a failed attempt at treatment, and the third case presents a patient’s refusal to get treated. Furthermore, a literature review highlighting other institutions’ attempts, successes, and failures at treating this clinical entity is presented. Combining the data retrieved from our institution and others, this review demonstrates that there is currently no standard for treating these patients. Additionally, based on the small case series and literature review presented in this article, definitive guidelines for the treatment of pseudoarthrosis of the distal humerus in pediatric OI patients cannot be outlined. However, our findings suggest that both non-surgical and surgical treatments could be viable options for patients with asymptomatic pseudoarthrosis of the distal humerus.

## Introduction

Osteogenesis imperfecta (OI), also known as brittle bone disease, is a rare skeletal disorder characterized by bone fragility leading to increased susceptibility to bone fractures [1]. The incidence of OI is estimated to be between 1/15,000 and 1/20,000 live births [2]. In 90% of patients, OI is due to a heterozygous mutation in either the *COL1A1* or *COL1A2* genes affecting collagen type 1 biosynthesis. Inheritance of these mutations follows an autosomal dominant pattern but can also arise sporadically [3]. In 1979, Sillence et al. developed a classification system for OI based on clinical manifestations of the disease, in which they divided their classification into types 1-4. Later, researchers discovered that OI was most commonly caused by mutations in *COL1A1/2* genes [4]. The remaining 10% of mutations responsible for OI are non-collagen mutations and have various inheritance patterns [3]. In 2004, Rauch and Glorieux expanded Sillence’s classification system adding types 5-7 with different inheritance patterns [5]. Since Rauch and Glorieux expanded the classification, multiple types of OI have been identified, with classification still a debated topic [6].

Due to its complexity, the management of OI in the pediatric population requires a multidisciplinary team working to reduce the likelihood of fractures and the development of deformities in patients [7]. The foundation of medical therapy for the management of OI is the use of bisphosphonates, physical therapy, and orthopedic interventions, such as intramedullary telescopic rods [8]. However, even with appropriate management, OI patients are still at risk of developing fractures and their associated complications. One such complication, which is more common in OI, is the development of a non-union leading to pseudoarthrosis [9]. Pseudoarthrosis is a manifestation of non-union, whereby an unhealed fracture structurally functions as a false joint. Non-unions in OI commonly occur after an acute fracture or realignment osteotomy [7]. Although it is difficult to estimate the incidence of this complication, Agarwal et al. found that non-union of long bones occurred in 18% of their pediatric population with OI over a 14-year period [10]. Non-union of a fracture can lead to deformity of the patient’s limb, pain, and instability, which, in turn, can limit the patient’s range of motion and greatly impact their quality of life [7].

Few articles in the literature have discussed the management of pediatric patients diagnosed with OI who experience a non-union in the distal third of their humerus. This article aims to summarize the currently published literature surrounding this topic and present three case reports of patients treated with this condition. In doing so, the purpose of this article is to demonstrate how pseudoarthrosis of the distal humerus in pediatric OI patients can be managed successfully via surgical treatment and conservative treatment in asymptomatic patients.

## Case presentation

The primary aim of the case presentations in this article is to elaborate on the challenges inherent in treating pseudoarthrosis of the distal humerus in pediatric OI patients. While each case delineates patient demographics, including age, OI type, presenting symptoms, previous treatment, surgical interventions, and ultimate outcomes, it is imperative to explicitly underscore the purpose of these clinical vignettes. Specifically, these cases serve to illustrate the diverse spectrum of clinical presentations, treatments, and functional outcomes encountered in the management of pseudoarthrosis of the distal humerus in OI patients.

Case 1

**Figure 1:**
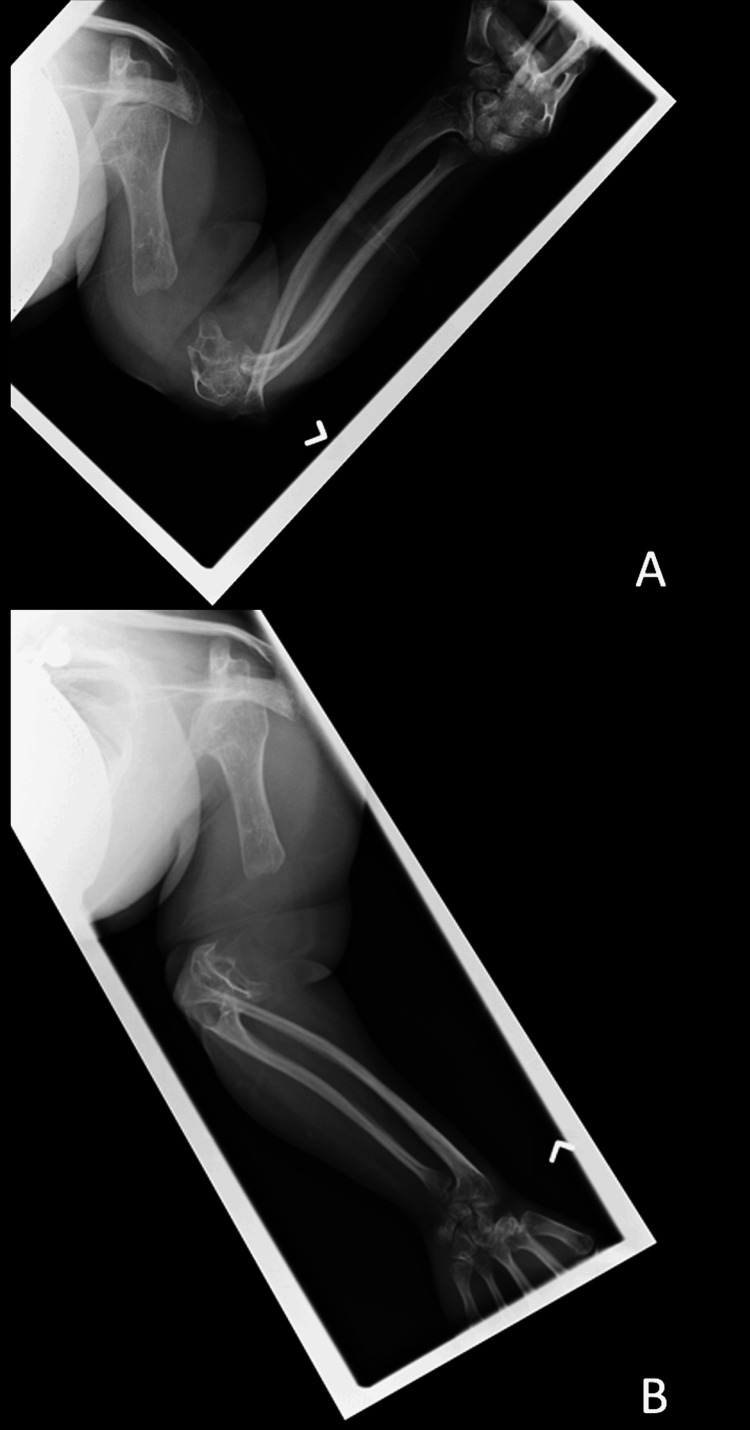
X-ray radiographs (A, B) showing a gap non-union of the distal left humerus in full extension and flexion at the elbow joint taken at 20 years old.

A female diagnosed with type 3 OI with a defined 4375 G --> C (Gly 1281 --> Arg) mutation in her *COL1A1* gene presented at age 15 complaining of a painful protrusion in her left distal humerus that was unsuccessfully operated on in the previous year for pseudoarthrosis. Imaging and physical examination revealed that the painful protrusion was a free-floating K-wire used to repair the pseudoarthrosis of her distal left humerus, causing a hematoma. The patient had the K-wire removed and was discharged from the hospital without complications. Six years later, at age 21, the option of repairing her pseudoarthrosis using a vascularized bone graft was proposed. The patient ultimately decided to not pursue the surgery as the pseudoarthrosis was not interfering with her quality of life. The patient is currently 31 years old and has been taking vitamin D and calcium since 1996. She uses a wheelchair to ambulate due to a spinal fusion in 2004 but can swim twice a week and lives an active professional life. The patient reports good mobility and no significant impairment regarding her pseudoarthrosis in her humerus. Furthermore, neurological examination demonstrates normal sensory and motor function in the affected and contralateral upper limbs (Figure 1).

Case 2

**Figure 2:**
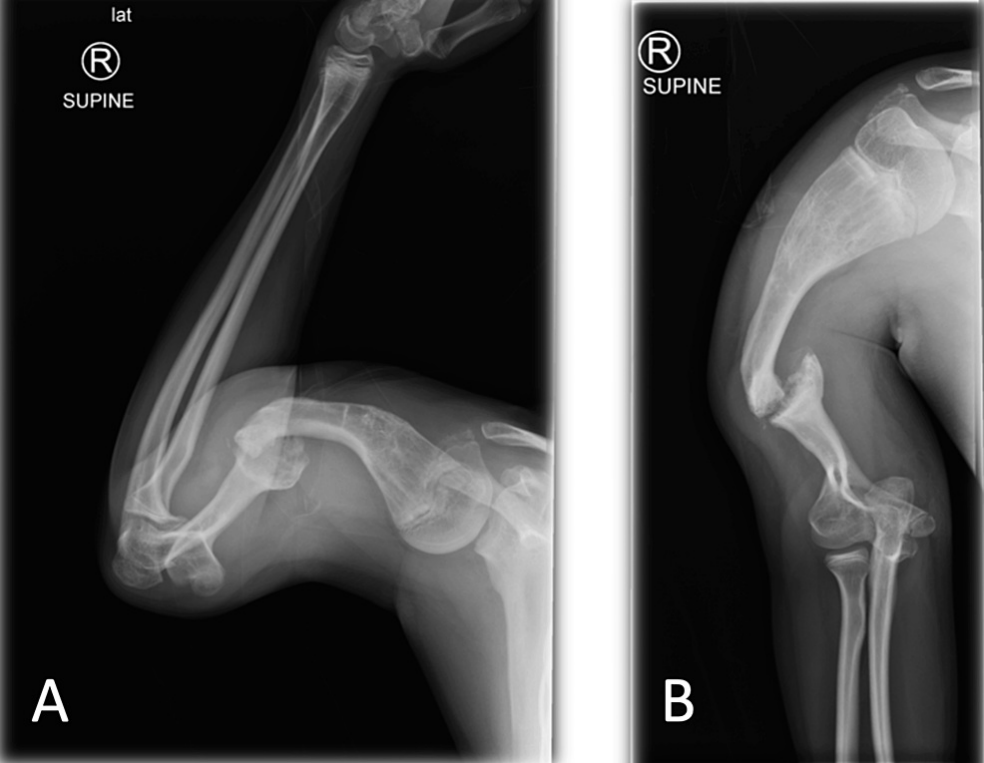
X-ray radiographs (A, B,) showing non-union and angular deformity of the right distal humerus in full extension and flexion at the elbow joint.

A 15-year-old male with type 4 OI with a heterozygous variant in exon 51 of *COL1A1* sustained a fracture to his right distal humerus. He was found to have developed pseudoarthrosis in the distal segment of his right humerus causing a deformity. On physical examination, the patient reported no pain with movement or palpation. Additionally, the patient was found to have a full range of motion in his shoulder, elbow, forearm, wrist, and fingers. Neurological examination was normal distal to the site of the deformity. The patient and his family decided not to proceed with surgery after discussing the risks with a surgeon. Ten months later, the patient was mobilizing well and did not present with any further concerns. Neurological examination of the contralateral and affected upper limbs of the patient showed normal sensory and motor function (Figure 2).

Case 3

**Figure 3:**
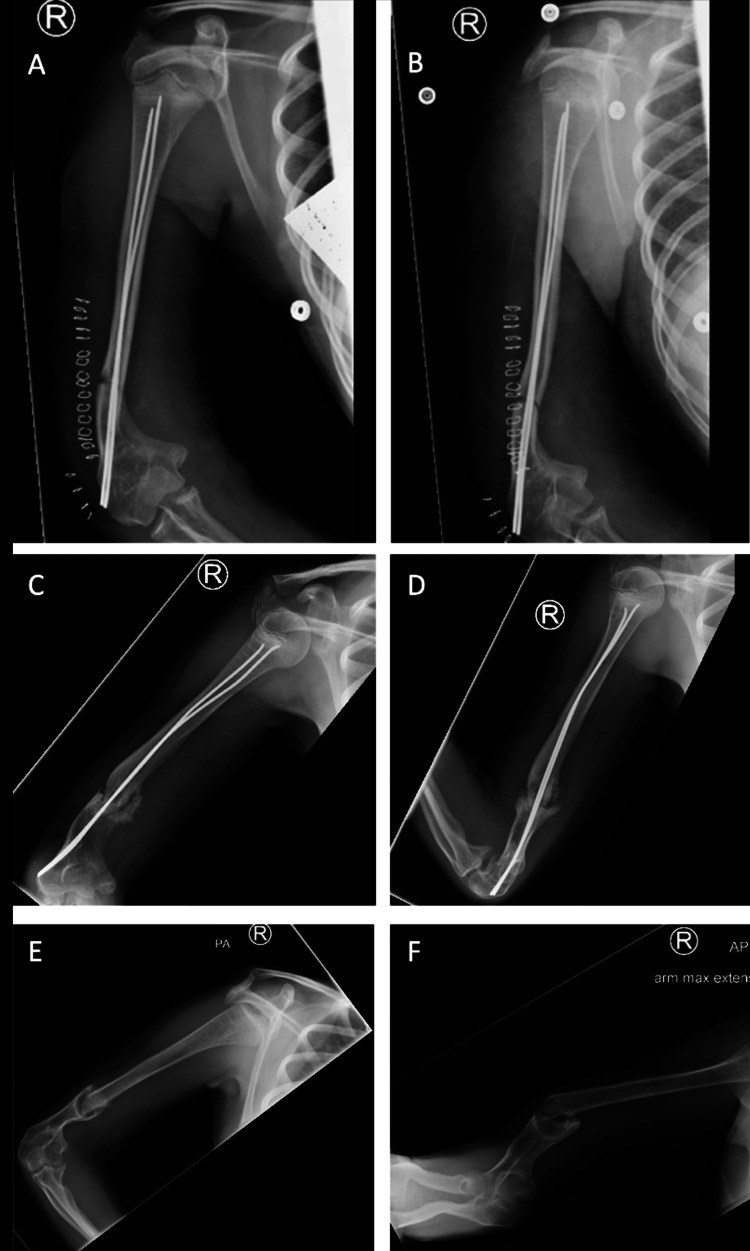
X-ray radiographs (A, B) showing postoperative placement of simple locking intramedullary nails in the right humerus, (C, D) non-union with hypertrophic features, and (E, F) deformity and non-union of the right distal humerus after removing nails causing skin irritation.

**Figure 4:**
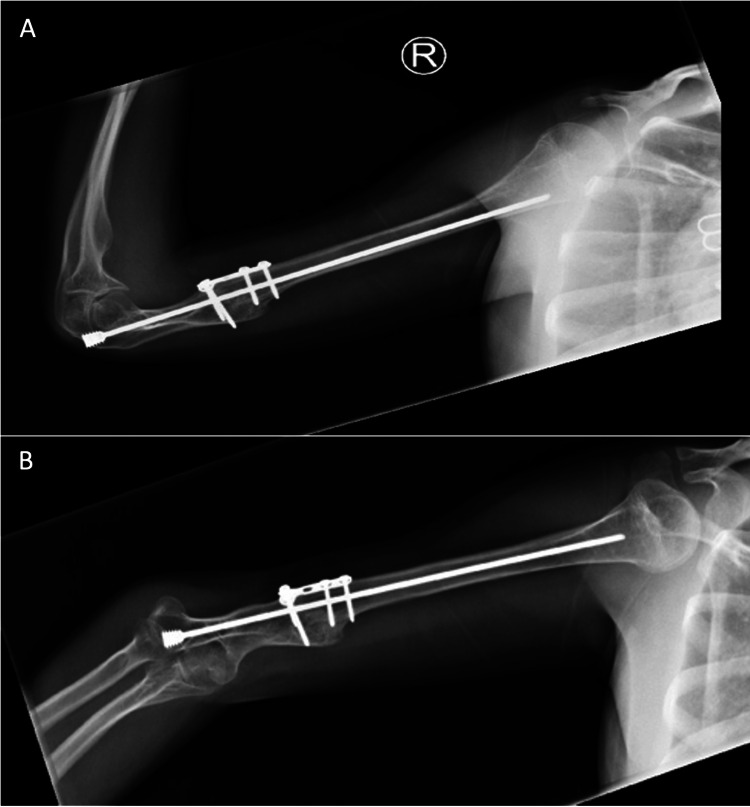
X-ray radiographs (A, B) showing union and complete healing of the distal right humeral non-union.

A 17-year-old female with type 3 OI caused by a heterozygous variant in exon 33 of *COL1A1* presented in the clinic two years after having suffered a fracture in her right humerus. Initially, her right humerus was treated by inserting retrograde titanium elastic nails. However, over the course of two years, the patient developed a hypertrophic non-union at the site of the fracture which led to the nails protruding and irritating her skin. This irritation caused the patient to return to the clinic at the age of 17 where the nails were surgically removed. Subsequently, the patient developed pain, instability, and malalignment at the site of pseudoarthrosis. She underwent surgery in which the hypertrophic non-union tissue was removed, a simple locking intramedullary (SLIM) nail was inserted in a retrograde fashion, and a demineralized bone matrix with a locking plate was inserted. The patient was seen one year later and showed signs of complete radiographic union. Furthermore, neurological examination demonstrated normal sensory and motor function in the affected and contralateral upper limbs (Figures 3, 4).

## Discussion

This literature review highlights the current challenges faced by clinicians when treating pseudoarthrosis of the distal humerus in the pediatric population diagnosed with OI. Although various surgical methods have been tested when attempting to correct non-union in the distal humerus of OI patients, the results of this literature review indicate that there is no consensus on the best method to treat this condition at present. This causes many patients to undergo multiple corrective surgeries without guaranteeing success, which, in turn, can cause significant stress and decreased quality of life for both the patient and their family. Furthermore, surgical correction of non-union is associated with multiple complications such as non-union [11,12].

Electronic databases PubMed, Ovid Medline, Ovid All EBM Reviews, Ovid Embase, and Web of Science were searched in July 2023. No publication date or type restrictions were utilized, and only English-language manuscripts were analyzed. The following keywords were applied as MeSH terms: “Osteogenesis imperfecta,” “humerus,” “non-union,” “nonunion,” “pseudoarthrosis,” “distal humerus,” “humeral fracture,” “nonunionated,” and “non-unionated.” A total of 3,374 articles were retrieved. Screening of the articles to include in this review was conducted by assessing article titles, with selection criteria defined by the presence of the keywords “humerus,” and “non-union,” or “pseudoarthrosis,” and “OI” or “osteogenesis imperfecta,” in the article titles, resulting in the inclusion of six articles. Table 1 summarizes the results of these articles, with further details in the Appendices (Table 2).

**Table 1:** Review of the literature surrounding the surgical management of pseudoarthrosis of the distal humerus in the pediatric osteogenesis imperfecta population. y/o = year old; BMP = bone morphogenic protein

Authors	Patient	Site	Surgery	Outcome
Agarwal et al., 2005 [10]	6 y/o, M	Left distal humerus	Telescoping rod + bone graft	No union
Agarwal et al., 2005 [10]	13 y/o, M	Right distal humerus	Rush rods + none graft	No union
Hsiao et al., 2013 [11]	2 y/o, M	Right distal humerus	Fassier-Duval nail + plate fixation + BMP	Union
Gamble et al., 1988 [13]	4 y/o, F	Left distal humerus	Bailey-Dubow rod + bone graft	Union
Gamble et al., 1988 [13]	22 y/o, F	L/R distal humerus	N/A	No union
Gamble et al., 1988 [13]	8 y/o, M	Right distal humerus	Schneider rod + bone graft	Union
Raut et al., 1987 [14]	11 y/o, M	Right distal humerus	Rush nail + bone graft	No union
Franzone et al., 2018 [15]	1 patient	Distal humerus	Telescoping rod + plate fixation	Union
Ashby et al., 2018 [16]	4 patients	Distal humerus	Revision rodding + bone graft	Union and non-union

Within the six articles identified in this review, the authors reported on 12 patients. Six of the 12 non-unions (50%) included in this review achieved radiographic union [11,13,15,16]. Union in the distal third of the humerus was attained using various surgical techniques. The main surgical method was the use of intramedullary rodding using devices such as Fassier-Duval, Bailey-Dubow, and Schneider rods. Supplemental locking plates, nails, bone morphogenic protein, and bone grafts were also utilized to achieve union [11,13,15,16]. One patient with a painless non-union in both right and left humeri accepted her condition and opted not to undergo surgery; instead, she was given bilateral containment orthoses and discharged [13]. Although it was reported that certain patients had up to three surgeries when trying to achieve union, four of the six patients who achieved union did so after one surgery, and the remaining two achieved union after two surgeries [11,13,15,16]. Although this review included very few patients, the previously stated remark may indicate that after multiple attempts at surgical correction of pseudoarthrosis in the distal humerus, there is no increased chance for union. However, further studies must be undertaken to confirm this. Nine of the 12 patients underwent treatment that utilized bone grafts; however, only four of the nine patients achieved bony union [10,13,14,16]. The two patients who received non-autogenous grafts failed to unite [10].

Despite poor results, it is widely accepted that the current gold standard for treating patients with OI and long bone fractures is intramedullary fixation [11]. Several modifications to this technique have been made, showing promising results. Hsiao et al. reported a successful case of distal humerus union in an OI patient treated with intramedullary fixation, locking plates, and infusion of bone morphogenic protein after a corrective surgery [11]. Similarly, Franzone et al. achieved union using intramedullary fixation and locking plates, adding additional locking plates in a second corrective surgery [15]. Likewise, Gamble et al. successfully treated this condition by employing Schneider and Bailey-Dubow rods as intramedullary fixators of the humerus and using local bone grafts [13].

Although not fully understood, non-union occurrence in patients with OI is a common complication. Agarwal et al. reported non-unions in 18% of children with OI who had long bone fractures [10]. Several reasons support the high incidence of non-union in these patients’ humerus. Internal fixation of a non-union may be difficult to achieve due to the decreased bone density of these patients [12]. Moreover, the distal humerus is subjected to many angular and rotational forces that cannot be contained by traditional casting and internal fixation [11]. Furthermore, the distal humerus has been shown to contain a “watershed” area with reduced blood supply that is prone to failed bone healing. This area may contribute to the increased incidence of non-union at the level of the distal humerus [12].

Surgical intervention for the treatment of distal humerus non-union in OI patients may be considered the gold standard [11]; however, it is important to be aware of another option for treatment, which is simply to brace the affected area. Gamble et al. reported on a patient with asymptomatic bilateral humeral non-union treated successfully with containment orthosis [13]. Similarly, Zionts et al. recommend not to surgically treat patients with asymptomatic non-unions of the upper limb but rather treat them using braces [7]. Agarwal et al. further support restricting surgery to symptomatic patients, stating that non-unions resulting from corrective osteotomy do not usually lead to disability and can be followed without surgical intervention if the patient can function with a normal quality of life and deformity does not develop [10].

This study has several strengths. It serves to highlight current gaps in knowledge pertaining to the treatment of this condition. It presents our institution’s past methods of attempting to treat this vexing surgical problem. The cases dealt with at our institution add to the growing literature showing that certain patients (case one and case two) can live with pseudoarthrosis in their distal humerus without any severe compromise in their quality of life. Furthermore, we present one successful attempt at healing non-union in this population using SLIM nails in a retrograde fashion and locking plates combined with demineralized bone matrix. This adds to the current literature, which is still in search of a gold standard for the treatment of non-union in the distal humerus of OI patients.

This study also has limitations. Although this condition is very frustrating to treat, there is limited literature regarding its management. Furthermore, within the published literature, there is no consensus on what information should be included in reports of successful or non-successful treatments in this patient population; for example, type of OI, location of the non-union (R/L), time of immobilization, and time to a radiographic union are not all universally reported. Therefore, certain analyses within this literature review are incomplete, and conclusions are not as strong. Additionally, one notable limitation of this article is the small sample size. Due to the rarity of pediatric patients with OI presenting with pseudoarthrosis of the distal humerus, there were limited patients to report. As a result, the generalizability of the conclusions of this article may be limited.

In summary, pseudoarthrosis of the distal humerus in pediatric patients living with OI is a very challenging clinical entity to manage and there is currently no standardized method for operating on patients with this condition. Within our institution, we have had one success in treating this condition using SLIM nails and supplemental plate fixation; however, we have also had multiple failed attempts at union. These failed attempts have allowed us to monitor patients living with non-unions for up to 16 years and we have observed that patients with this deformity can live with a good quality of life without surgical intervention.

## Conclusions

By combining the data retrieved from our attempts at management as well as other institutions’ attempts at management, we have determined that there is currently no standard to treat pseudoarthrosis of the distal humerus in pediatric OI patients. Due to the limited amount of literature surrounding this topic, as well as our limited experience with this clinical entity, no definite conclusions can be made on how to treat pseudoarthrosis of the distal humerus in pediatric OI patients. However, surgeons and treating physicians should be aware of the possible outcome of the failure of surgical treatment and the high rate of persistent pseudoarthrosis. Furthermore, this article served to highlight that an asymptomatic pseudoarthrosis may be best treated non-operatively. Several avenues for future research warrant exploration, including studying the long-term effects of conservative treatment for asymptomatic pseudoarthrosis including radiographic and functional progression. Furthermore, attempts at repeating the successful SLIM nail surgical technique highlighted in the case series should be undertaken.

## Appendices

**Table 2:** Detailed review of the literature surrounding the surgical management of pseudoarthrosis of the distal humerus in the pediatric osteogenesis imperfecta population. OI = osteogenesis imperfecta; BMP = bone morphogenic protein

Authors and year	Patient	Deformity	OI type	R/L Side	Site	1st surgery	2nd surgery	3rd surgery	Bone Graft	Orthosis	Outcome
Agarwal et al., 2005 [10]	Six-year-old male	Gap non-union	N/A	Left	Junction of the middle and lower third of the humerus	A bone segment grafted from the patient’s right humerus was used to fill the gap non-union and a telescoping rod was inserted. Union failed in three months	A telescoping rod with an autogenous cortico-cancellous iliac bone graft was used; shoulder spica was applied, but the graft resorbed within two months	Telescoping rod and Orthofix external fixator were applied; graft was harvested from the mother’s tibia and fibula; shoulder immobilized in spica, and fixator was removed in 3 months due to loose screw; six months later, the rod was removed due to infection	1: Autogenous right humerus. 2: Autogenous cortico-cancellous iliac. 3: The mother’s tibia and fibula	Shoulder spica	Failed union
	13-year-old male		N/A	Right	The lower third of the humerus	Internal fixation using a Rush rod. Autogenous iliac bone grafting was performed. Eight months of cast immobilization	Specially designed Rush rods with terminal threads for fitting nuts. The bone was harvested from the patient’s father’s ilium as a graft; spica was used and pamidronate was given (×4 cycles)		1: Autogenous iliac. 2: Father’s ilium	Shoulder spica	Failed union
Ashby et al., 2018 [16]	Four patients		N/A	N/A	Distal humerus (two following osteotomy and two following fracture)	Revision rodding was used in all patients in addition to bone grafting	N/A	N/A	Bone graft	N/A	Bony union was achieved in the two cases where osteotomy sites had previously not healed. The fracture non-union sites did not heal despite multiple surgical procedures, including the use of bone morphogenic protein
Franzone et al., 2018 [15]	One patient		N/A	N/A	Distal humerus	Intramedullary fixation using a male component of a Fassier-Duval telescopic rod and a six-hole 2.4 mm locking compression plate with four locking screws and one cortical screw	Supplemental plate fixation was applied to the site of a non-union	N/A	N/A	N/A	Union
Gamble et al., 1988 [13]	Four-year-old female	Hypertrophy	Type 3	Left	Junction of the middle and distal one-third of the diaphysis	Male Bailey-Dubow rod and local bone graft	N/A	N/A	Local bone	N/A	Union after eight weeks
	22-year-old female	Atrophy (L), hypertrophy (R)	Type 3	Left and right	Junction of the middle and distal one-third of the diaphysis	No procedure was done	N/A	N/A	N/A	N/A	The patient uses bilateral containment orthoses
	Eight-year-old male	Hypertrophy	Type 3	Right	Junction of the middle and distal one-third of the diaphysis	Schneider rod and local bone graft	N/A	N/A	Local bone graft	N/A	Union after six weeks
Raut et al., 1987 [14]	11-year-old male		Type 3	Right	Distal humerus	A Rush nail was inserted and a one-month-old wedge resection graft from the patient’s left femur; placed in a shoulder spica	N/A	N/A	Autogenous 1-month-old femur graft	Shoulder spica	Non-union (the patient was discharged with a leather corset as permanent arthrosis)
Hsiao et al., 2013 [11]	Two-year-old male	Atrophic and varus angulation	Type 3	Right	Distal humerus	Intramedullary fixation with Fassier-Duval nail in addition to tricalcium sulfate (VI-TOSS). Fracture brace and ultrasonic bone stimulator 20 minutes/day; After five months, the fracture persisted	Revised intramedullary telescoping Fassier-Duval nail in addition to excision of the non-union and internal fixation with medial and lateral locked plating. The procedure was supplemented with infused BMP; six weeks of casting was applied	N/A	N/A	Six weeks of casting with a custom-fitted fracture brace	Union: radiographic healing after three months and follow-up at 25 and 34 months show complete union
